# Up‐regulation of HOXA‐AS2 and MEG3 long non‐coding RNAs acts as a potential peripheral biomarker for bipolar disorder

**DOI:** 10.1111/jcmm.70150

**Published:** 2024-11-01

**Authors:** Maryam Hosseini, Mohammad Javad Mokhtari

**Affiliations:** ^1^ Department of Biology, Zarghan Branch Islamic Azad University Zarghan Iran

**Keywords:** BD, biomarker, gene expression, LncRNA, real‐time PCR

## Abstract

Bipolar disorder (BD) is a psychiatric condition that is frequently misdiagnosed and linked to inadequate treatment. Long non‐coding RNAs (lncRNAs) have lately gained recognition as crucial genetic elements and are now regarded as regulatory mechanisms in the neurological system. Our objective was to measure the quantities of HOXA‐AS2 and MEG3 ncRNA transcripts. HOXA‐AS2 and MEG3 ncRNA levels were checked in the peripheral blood of 50 type I BD and 50 control samples by real‐time PCR. Furthermore, we conducted ROC curve analysis and correlation analysis to examine the association between gene expression and specific clinical characteristics in instances with BD. Additionally, a computational study was performed to investigate the binding sites of miRNAs on the HOXA‐AS2 and MEG3 lncRNAs. BD subjects showed a significant increase in the expression of HOXA‐AS2 and MEG3 compared to controls. The lncRNAs HOXA‐AS2 and MEG3 have an area under the ROC curve (AUC) values of 0.70 and 0.71, respectively. There was a significant correlation between the expression levels of ncRNAs HOXA‐AS2 and MEG3 in the peripheral blood of patients with BD and occupation scores. The data presented indicate a potential correlation between the expression of HOXA‐AS2 and MEG3 lncRNAs with an elevated risk of BD. Furthermore, these lncRNAs may be linked to several molecular pathways. Our findings indicate that the amounts of lncRNAs HOXA‐AS2 and MEG3 in transcripts might be a promising potential biomarker for patients with BD.

## 1 INTRODUCTION

Bipolar disorder (BD) is a highly debilitating and persistent mental disease that impacts roughly 1.06% of the population over their lifetime, namely type I BD.^1^ The cause of BD is not well understood; however, environmental and genetic variables are believed to have a role in its development.^2^ On the other hand, BD exhibits a wide range of clinical variations, making it challenging to identify and diagnose accurately.^3^ Furthermore, there is no complete answer to the available treatments. Hence, it is imperative to conduct neurobiological research to discover readily available molecular biomarkers in peripheral blood, which would ultimately contribute to the diagnosis and efficient treatment of BD.^4^ Multiple expression profiling studies have shown that abnormal ncRNA expression is implicated in a range of biological and pathogenic processes, such as Alzheimer's disease (AD),^5^ schizophrenia,^6^ depression,^7^ and BD.^8^ Although the biological mechanisms of BD are poorly understood, studies suggest that dysregulated gene expression may make an intriguing contribution to the development of BD. Altered expression of several long noncoding RNAs (lncRNAs) such as DISC1, DISC2, RMRP, CTC‐487 M23.5, DGCR5, MALAT1, SCAL1, RMST, MEG3, lincRNA‐p21, lincRNA‐ROR and lincRNA‐PINT was observed in BD patients.^8^, ^9^, ^10^, ^11^ In this research, we employed a literature‐based methodology to discover lncRNAs that may have a potential but indirect or inadequately assessed involvement in BD. It is very important to identify more new BD‐related lncRNAs and explore their biological functions in order to explore new therapeutic methods and potential diagnostic biomarkers for BD. We chose two lncRNAs, namely HOXA‐AS2 and MEG3, to evaluate their expression in the peripheral blood of BD patients and healthy subjects. The expression, function, and role of HOXA‐AS2 in BD are still not well studied.

HOXA‐AS2 is produced by transcription from the HOXA cluster, specifically in the region between and in the opposite direction of the human HOXA3 and HOXA4 genes.^12^ Given the significance of the HOXA gene in the development of the nervous system and the connection between improper brain development and the development of schizophrenia, HOXA‐AS2 may have a role in this mental disorder.^6^ In addition, HOXA‐AS2 has a significant impact on the polarization of microglial cells and is noticeably increased in PBMCs obtained from individuals with Parkinson's disease.^13^

MEG3, a gene expressed maternally, controls the production of AMPA glutamate receptors in early cortical neurons.^14^ MEG3 overexpression decreases neuronal activity and enhances cell apoptosis.^15^ MEG3 modulates hypoxia‐induced neuronal death via impacting lipoxygenase signalling, a mechanism discovered to be impaired in the brain tissues of individuals with BD.^16^, ^17^ MEG3 plays a role in regulating oxidative stress.^18^

While the functions of HOXA‐AS2 and MEG3 have been shown, their precise molecular mechanisms in the development of BD have not been studied. Consequently, identifying their expression as molecular biomarkers should enhance our comprehension of the underlying causes of BD. This research aimed to evaluate the differential expression of lncRNA genes HOXA‐AS2 and MEG3 in the peripheral blood of patients diagnosed with BDI compared to a control group of healthy individuals. Therefore, the abnormal regulation of specific ncRNAs may have a role in the development of BD or serve as a diagnostic indicator for the disease.

## 2 MATERIALS AND METHODS

### 2.1 Study subjects

**FIGURE 1:**
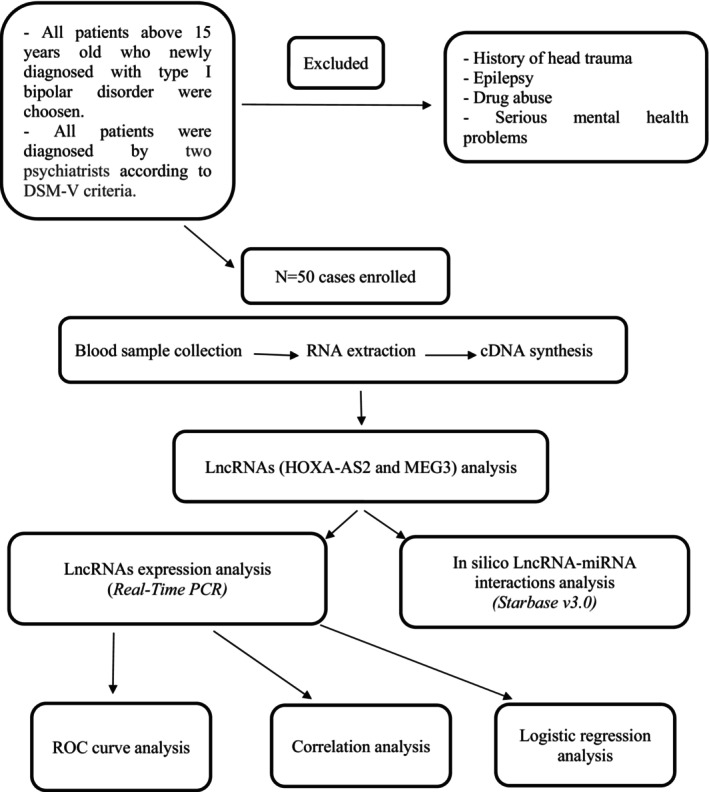
Flowchart of data collection and method implementation.

In this research, 50 individuals with BD type I and 50 controls were selected. No significant difference was shown in age and sex ratio (*p >* 0.05). All patients in this study were diagnosed by two psychiatrists according to DSM‐V criteria. They referred to the Ibn‐e‐Sina Hospital in Shiraz, Iran. We applied the following characteristics as exclusion criteria: history of head trauma, epilepsy, drug abuse and severe mental health problems. Controls were randomly selected from the same source population as the BD type I patients. The subjects examined were aged 15–65 years. The Ethics Committee of the Arsanjan Branch, Islamic Azad University, Iran, approved this study (IR.IAU.A.REC.1399.027). The participants provided written informed consent and willingly accepted to participate in the current investigation. Figure 1 shows the study's flowchart of data collection and method implementation.

### 2.2 RNA extraction, cDNA synthesis and real‐time PCR

Total RNA was extracted from whole blood using a complete RNA extraction kit (Favorgen, Taiwan). Then, cDNA was synthesized using a cDNA synthesis Kit (Yektatajhiz, Iran). Primer Express v.3.0 was used to design the primers. Specific primers of HOXA‐AS2, MEG3, and the *B2M* gene (used as the endogenous normalizer real‐time PCR) are shown in Table 1. We utilized the Quantstudio 3 real‐time PCR instrument (manufactured by Applied Biosystems, USA) with a thermal cycling protocol consisting of a 5‐min incubation at 95°C (one repetition), followed by 40 cycles of 15 s at 95°C and 1 min at 60°C. Every complete amplification stage is followed by a melting phase consisting of three temperature steps: 15 s at 95°C, 30 s at 60°C, and another 15 s at 95°C. Each relative expression was determined using the 2^−ΔCt^ strategy.^19^

**TABLE 1:** The nucleotide sequence of primers.

Gene name	Primer sequences	Primer length (bp)	Product length (bp)
HOXA‐AS2	*CCCGTAGGAAGAACCGATGA*	20	70
	*TTTAGGCCTTCGCAGACAGC*	20	
MEG3	*TGGCATAGAGGAGGTGAT*	18	111
	*GGAGTGCTGTTGGAGAATA*	19	
B2M	*AGATGAGTATGCCTGCCGTG*	20	105
	*GCGGCATCTTCAAACCTCCA*	20	

### 2.3 In silico LncRNA‐miRNA interactions analysis

The online prediction software Starbase v3.0 (starbase.sysu.edu.cn/starbase2/index.php) was employed to investigate the binding of HOXA‐AS2 and MEG3 lncRNA to miRNAs. This analysis aimed to find the miRNAs that can lncRNA. StarBase v3.0 mainly identifies RNA–RNA and protein–RNA interaction networks in CLIP‐Seq (cross‐linking immunoprecipitation) datasets.^20^ In order to forecast the miRNA target sites, the CLIP data was set to strict stringency (≥5).

### 2.4 Statistical analysis

GraphPad Prism 5.0 and SPSS software (version 22.0) were used for the analyses. The gene expression level was reported as the mean value plus or minus the SE. The mean expression of each gene was compared between case groups and healthy controls using the Student's *t*‐test. Receiver operating characteristic (ROC) curve analysis was conducted to determine the diagnostic potential of HOXA‐AS2 and MEG3 levels as biomarkers for type I BD, based on the area under the curve (AUC). A Spearman's correlation coefficient was employed to assess the association between gene expression levels and clinical or demographic factors. Additionally, logistic regression analysis was conducted to evaluate the correlation between HOXA‐AS2 and MEG3 and the expression of BD, using the odds ratio as the measure of association. A *p*‐value < 0.05 was deemed statistically significant.

## 3 RESULTS

### 3.1 Demographic data

The study comprised a cohort of 50 participants diagnosed with BDI. The control group comprised 50 individuals who were healthy and matched in terms of age, sex and ethnicity. Significant differences were seen between the BDI group and the control group regarding education, job, and marital status (*p* < 0.05). Table 2 provides a summary of the demographic features of both groups.

**TABLE 2:** Demographic characteristics of the BD patients and controls.

Characteristics	Cases (*n* = 50)	Controls (*n* = 50)	*p*‐value
Male, *n* (%)	18 (36%)	19 (38%)	0.83^a^
Female, *n* (%)	32 (64%)	31 (62%)	
Age, years	37.96 ± 1.64	41.42 ± 1.57	0.13^b^
Education			
School	36 (72%)	22 (44%)	0.005^a^
University	14 (28%)	28 (56%)	
Job			
Practitioner	18 (36%)	28 (56%)	0.045^a^
Unemployed	32 (64%)	22 (44%)	
Marital status			
Married/cohabitating	28 (56%)	42 (84%)	0.004^a^
Single	17 (34%)	8 (16%)	
Divorced	5 (10%)	–	

*Note*: Quantitative and qualitative data were shown as mean ± standard deviation (SD) and no. (%), respectively.

^a^ Independent two‐sample *t*‐test;

^b^ Chi‐square test.

### 3.2 LncRNAs expression analysis

**FIGURE 2:**
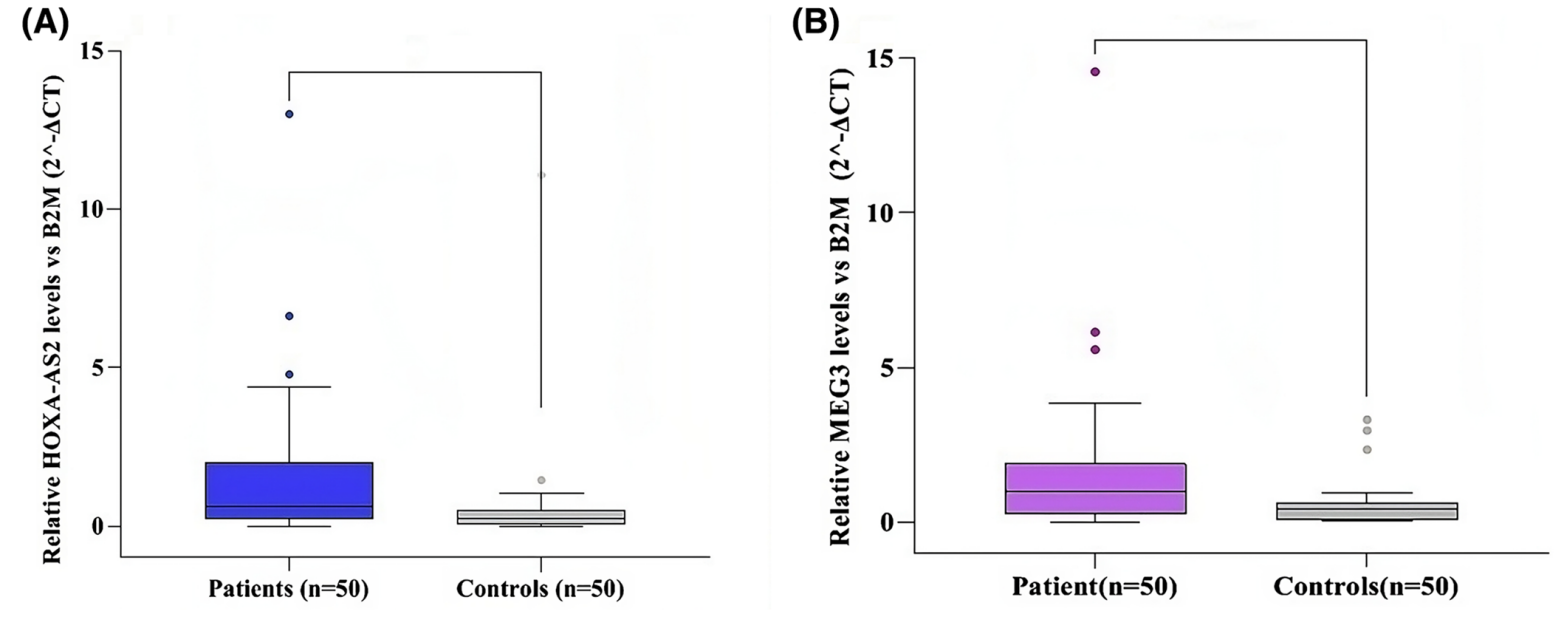
The expression of LncRNA HOXA‐AS2 (A) was up regulated significantly in BD patients and LncRNA MEG3 (B) was up regulated significantly in BD patients. The relative expression (fold change) of transcripts was calculated using the equation 2^−ΔCt^ with independent *t*‐test. The gene expression values of each sample were normalized relative to B2M expression as reference gene. Significance was set at *p < 0.05, **p < 0.001.

Figure 2 illustrates the comparative expression levels of HOXA‐AS2 and MEG3 in the peripheral blood of individuals with BD compared to healthy controls. The results revealed a substantial increase in the expression of HOXA‐AS2 (fold change 2.97, *p* = 0.003) and MEG3 (fold change 2.77, *p* = 0.015) in the peripheral blood of individuals with BD compared to the control group (Figure 2A,B). Consequently, there was a significant difference in the expression of HOXA‐AS2 between male individuals with BD and the control group (*p* = 0.014, 3.46‐fold change). Similarly, there was a significant difference in the expression of MEG3 between female individuals with BD and the control group (*p* < 0.001, 5.00‐fold change). The data is not displayed.

### 3.3 The ROC curve analysis

**FIGURE 3:**
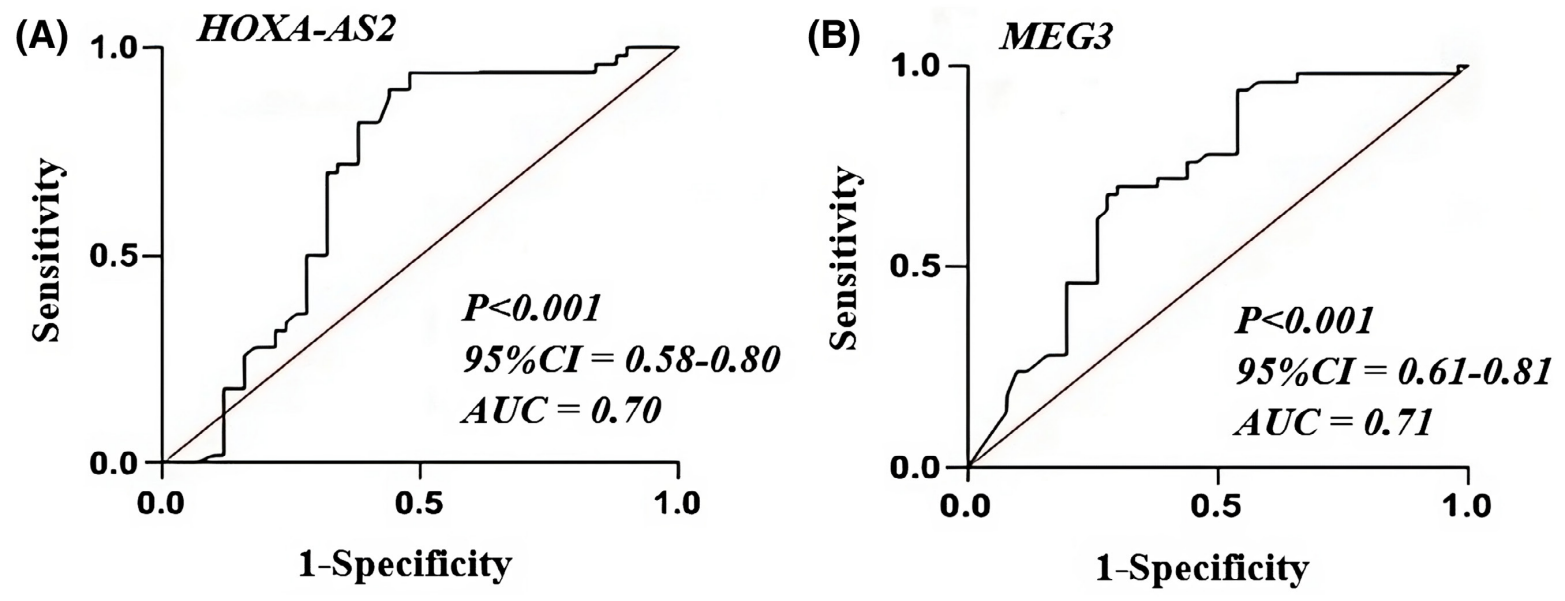
ROC curve analysis of HOXA‐AS2 (A) and MEG3 lncRNA (B) expression for differentiating BD patients from healthy controls. The sensitivity was plotted against the 1‐specificity for each threshold value. AUC, area under curve.

The specificity and sensitivity of HOXA‐AS2 and MEG3 lncRNA gene expressions were assessed using ROC analysis as possible biomarkers for the clinical diagnosis of BD. The AUC values for HOXA‐AS2 (Figure 3A) and MEG3 lncRNA (Figure 3B) were 0.70 (*p* < 0.001, 95% CI: 58%–80%) and 0.71 (*p* < 0.001, 95% CI: 61%–81%), respectively.

### 3.4 Correlation analysis

**FIGURE 4:**
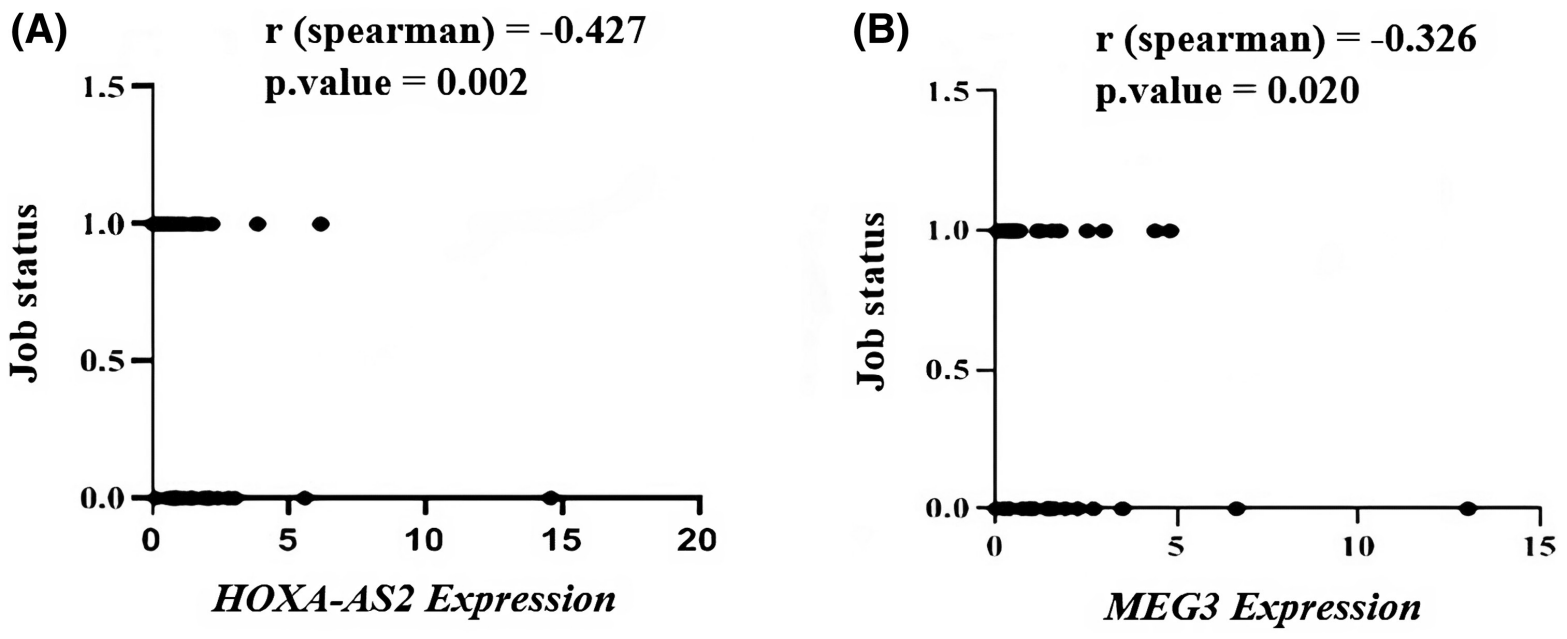
The correlation analysis between the expression levels HOXA‐AS2 and MEG3 lncRNA transcripts with job status of BD patients. (A) Correlation between HOXA‐AS2 levels and job status. (B) Correlation between MEG3levels and job status.

There was no statistically significant relationship found between the expression level of HOXA‐AS2 lncRNA in the peripheral blood of BD patients and their sex (*r* = 0.14, *p* = 0.31), age (*r* = 0.25, *p* = 0.07), marital status (*r* = −0.18, *p* = 0.19) and education (*r* = −0.19, *p* = 0.16). A significant positive correlation was observed between the expression of HOXA‐AS2 lncRNA and job status (*r* = −0.42, *p* = 0.002) (Figure 4A). In addition, there was no substantial association observed between the expression level of MEG3 lncRNA and sex (*r* = 0.03, *p* = 0.81), age (*r* = 0.07, *p* = 0.59), marital status (*r* = −0.05, *p* = 0.69) and education (*r* = −0.001, *p* = 0.99). A significant positive correlation was observed between the expression of MEG3 lncRNA and job status (*r* = −0.32, *p* = 0.02) (Figure 4B).

### 3.5 In silico LncRNA‐miRNA interactions analysis

miRNAs can target multiple lncRNAs. To predict the association between our RNA and miRNA, starbase v3.0. was employed. The variations in gene expression of HOXA‐AS2 and MEG3 may be attributed to interactions with various miRNAs, as miRNAs can regulate the expression of crucial genes involved in the development of BD. Hence, discovering the miRNA responsible for regulating the expression of these two genes should facilitate the identification of additional molecular pathways underlying BD illness. Starbase is a web service that allows for thorough exploration of miRNA‐target interaction maps using CLIP‐Seq and Degradome‐Seq data. HOXA‐AS2 may be regulated by hsa‐miR‐302e, hsa‐mir‐17‐5p, hsa‐mir‐20a‐5p, hsa‐miR‐520e, hsa‐miR‐520a‐3p, hsa‐miR‐520b, hsa‐miR‐520c‐3p, hsa‐miR‐519d‐3p, hsa‐miR‐520d‐3p, hsa‐miR‐372‐3p, hsa‐miR‐373‐3p, hsa‐miR‐302d‐3p, hsa‐miR‐302a‐3p, hsa‐miR‐302c‐3p, hsa‐miR‐302b‐3p, hsa‐miR‐93‐5p, hsa‐miR‐106b‐5p, hsa‐miR‐20b‐5p and hsa‐miR‐106a‐5p and MEG3 lncRNA may be controlled by hsa‐miR‐7‐5p, hsa‐miR‐4782‐3p, hsa‐miR‐219a‐5p and hsa‐miR‐361‐5p (Table 3). However, these two genes did not share any miRNA.

**TABLE 3:** miRNA target sites predicted using StarBase on H19 and MALAT1.

Gene	miRNA	Target site	Target sequence	miRNA sequence
HOXA‐AS2	hsa‐miR‐302e	chr7:27156121‐27156137[+]	acucacuuuAGCACUUu	uucguaccuUCGUGAAu
	hsa‐miR‐17‐5p	chr7:27156118‐27156138[+]	uggaCU‐CACU‐UUAGCACUUUc	gaugGACGUGACAUUCGUGAAAc
	hsa‐miR‐20a‐5p	chr7:27156118‐27156138[+]	uggaCU‐CACU‐UUAGCACUUUc	gaugGACGUGAUAUUCGUGAAAu
	hsa‐miR‐520e	chr7:27156117‐27156137[+]	auggacucacuuuAGCACUUu	gggaguuuuuccuUCGUGAAa
	hsa‐miR‐520a‐3p	chr7:27156116‐27156137[+]	aauggacucacuuuAGCACUUu	ugucagguuucccuUCGUGAAa
	hsa‐miR‐520b	chr7:27156117‐27156137[+]	auggacucacuuuAGCACUUu	gggagauuuuccuUCGUGAAa
	hsa‐miR‐520c‐3p	chr7:27156116‐27156137[+]	aauggacucacuuuAGCACUUu	ugggagauuuuccuUCGUGAAa
	hsa‐miR‐519d‐3p	chr7:27156117‐27156138[+]	auggacucacuuuaGCACUUUc	gugagauuucccucCGUGAAAc
	hsa‐miR‐520d‐3p	chr7:27156116‐27156137[+]	aauggacucacuuuAGCACUUu	ugggugguuucucuUCGUGAAa
	hsa‐miR‐372‐3p	chr7:27156115‐27156137[+]	uaauggacucacuuUAGCACUUu	ugcgaguuuacagcGUCGUGAAa
	hsa‐miR‐373‐3p	chr7:27156115‐27156137[+]	uaauggacucacuuuAGCACUUu	ugugggguuuuagcuUCGUGAAg
	hsa‐miR‐302d‐3p	chr7:27156119‐27156137[+]	ggACUC‐‐ACUU‐‐UAGCACUUu	ugUGAGUUUGUACCUUCGUGAAu
	hsa‐miR‐302a‐3p	chr7:27156115‐27156137[+]	uaAUGGACUCACUUUAGCACUUu	agUGGUUUUGUACCUUCGUGAAu
	hsa‐miR‐302c‐3p	chr7:27156115‐27156137[+]	uaAUGGACUCACUUUAGCACUUu	ggUGACUUUGUACCUUCGUGAAu
	hsa‐miR‐302b‐3p	chr7:27156115‐27156137[+]	uaAUGGACUCACUUUAGCACUUu	gaUGAUUUUGUACCUUCGUGAAu
	hsa‐miR‐93‐5p	chr7:27156113‐27156138[+]	cguaaUGGACUCACUUUAGCACUUUc	gauggACGUGCUUG‐‐‐UCGUGAAAc
	hsa‐miR‐106b‐5p	chr7:27156120‐27156138[+]	gaCU‐CACU‐UUAGCACUUUc	uaGACGUGACAGUCGUGAAAu
	hsa‐miR‐20b‐5p	chr7:27156118‐27156138[+]	uggaCU‐CACU‐UUAGCACUUUc	gaugGACGUGAUACUCGUGAAAc
	hsa‐miR‐106a‐5p	chr7:27156118‐27156138[+]	uggaCU‐CACU‐UUAGCACUUUc	gaugGACGUGACAUUCGUGAAAa
MEG3	hsa‐miR‐7‐5p	chr14:101297777‐101297798[+]	ugucUACACUUGCU‐GUCUUCCu	uguuGUUUUAGUGAUCAGAAGGu
		chr14:101300910‐101300932[+]	guugccuucUUCCUCGUCUUCCu	uguuguuuuAGUGAUCAGAAGGu
	hsa‐miR‐4782‐3p	chr14:101296962‐101296982[+]	uuUCUGGG‐GCACCGACAAUCu	caAGAUCUAUACUUCUGUUAGu
	hsa‐miR‐219a‐5p	chr14:101296962‐101296982[+]	uuucuggggcaccGACAAUCu	ucuuaacgcaaacCUGUUAGu
	hsa‐miR‐361‐5p	chr14:101296988‐101297012[+]	guGCACCAGGCUAGGUAUCUGAUAc	caUGGGGACC‐‐UCUA‐AGACUAUu

### 3.6 Logistic regression analysis

Table 4 presents the results of logistic regression analysis. Results demonstrate that HOXA‐AS2 upregulation is a significant risk factor for BD. Also, a significant association was observed about the age of individuals. However, no significant association was observed concerning gender individuals.

**TABLE 4:** Odd ratio result of HOXA‐AS2, MEG3, sex and age.

	HOX expression	MEG3 expression	Sex	Age
OR	2.72	1.148	1.05	0.950
P‐value	0.005	0.496	0.905	0.014
(95% CI)	1.35–5.50	0.771–1.70	0.423–2.639	0.912–0.990

*Note*: OR: Adjusted odds ratio is calculated for HOXA‐AS2 and MEG3 expression to determine the expression power of the genes in bipolar disorder (BD).

## 4 DISCUSSION

Multiple studies have investigated biomarkers associated with BD, including miRNA expression. miRNAs including miR‐15b and miR‐652,^21^ up‐regulated miR‐21‐3p, miR‐140‐3p as well as miR‐330‐5p in peripheral tissues^22^ and up‐regulated miR‐17‐5p, miR‐29c and miR‐29c‐3p in postmortem tissue^23^ involve in the pathogenesis of BD. These miRNAs contribute to the progression of BD. These findings suggest an additional instance of non‐coding RNA, precisely long lncRNA, which could significantly influence the advancement of BD.^10^, ^24^ In this investigation, we reported a significant up‐regulation of HOXA‐AS2 and MEG3 in individuals with BD as compared to healthy individuals. Furthermore, there was a significant difference in HOXA‐AS2 expression between male individuals with BD and the control group. Similarly, there was a difference in MEG3 expression between female individuals with BD and the control group. Sex‐based differences are evident in BD, which suggests that they could offer a fresh outlook on the underlying mechanisms of this condition in both males and females.^10^, ^23^, ^24^ The examination of the ROC curve demonstrated that the AUCs for HOXA‐AS2 and MEG3 were 0.70 and 0.71, respectively. This indicates that these genes can differentiate between individuals with BD and healthy controls.

The specificity and sensitivity of HOXA‐AS2 and MEG3 lncRNA gene expression were evaluated as potential biomarkers for clinical diagnosis of BD. Recently, it has been demonstrated that lncRNAs are effective diagnostic and prognostic biomarkers in various disorders.^25^, ^26^ LncRNAs are promising molecular biomarkers that may improve the reliability, sensitivity, and specificity of molecular techniques in clinical diagnosis due to their expression specificity in various diseases and stability in body fluids. The development of lncRNA‐based diagnosis and lncRNA‐based therapy will be useful in daily medical practice to improve clinical treatment and quality of life of patients.

In this study, we used computational methods to predict the interaction between lncRNAs and miRNAs. About 10,000 competetive endogenous RNAs (ceRNAs) pairs from CLIP‐supported miRNA target sites were identified in StarBase.^27^ Specifically, we found that hsa‐miR‐17‐5p, hsa‐miR‐20a‐5p, hsa‐miR‐520c‐3p, hsa‐miR‐106b‐5p and hsa‐miR‐106a‐5p have binding sites on HOXA‐AS2. Several studies have investigated the expression of these miRNAs in BD patients. Miller et al. examined the expression of 854 miRNAs in individuals with schizophrenia and BD.^28^ The expression of miR‐17‐5p, miR‐20a‐5p, hsa‐miR‐520c‐3p, hsa‐miR‐106b‐5p and hsa‐miR‐106a‐5p were significantly altered in BD patients.^29^, ^30^, ^31^ Also, we observed that hsa‐miR‐7‐5p, hsa‐miR‐4782‐3p, hsa‐miR‐219a‐5p and hsa‐miR‐361‐5p have binding sites on MEG3. Lee et al. reported that significantly elevated serum levels of miR‐7‐5p in BD patients than controls.^32^ However, to the best of our knowledge, there are no studies on the expression of hsa‐miR‐4782‐3p, hsa‐miR‐219a‐5p and hsa‐miR‐361‐5p in patients with BD.

Development of computational models is a novel strategy for identification of lncRNA–miRNA regulatory network of human complex diseases in BD. Several studies have demonstrated the importance of computational models in identifying novel miRNA‐disease associations, which may lead to the selection of important miRNA‐disease pairs for further experimental studies.^33^, ^34^ In a study by Huang et al., a multi‐view multi‐task miRNA‐disease association prediction model was used to calculate predictions for lncRNA‐miRNA pairs, where miRNAs were represented in both the microRNA‐disease association network view and the lncRNA‐miRNA interaction network view.^35^ The Laplace regularized least squares approach to determine lncRNA‐disease associations also led to the identification of several disease‐lncRNA associations.^36^ Similar strategies can be applied to discover biomarkers for BD. Several novel computational models have been used to classify disease‐associated lncRNAs on a large scale and select the most appropriate disease‐associated lncRNAs.^37^ It is noteworthy that the development of systematic functional annotation methods is crucial to improve the prediction accuracy of computational models and further accelerate the process of documenting novel lncRNA functions.

HOXA‐AS2 has demonstrated an interaction with the enhancer of zeste homologue 2 (EZH2). EZH2 plays a crucial role in the epigenetic silencing of cyclooxygenase‐2,^38^ an enzyme that is overexpressed in schizophrenia and BD as a result of immune response dysregulation.^39^ Also, schizophrenia is associated with increased levels of HOXA‐AS2 expression.^6^ Both manic and depressive BD episodes are accompanied by the activation of neuroinflammation pathways, as indicated by a positive profile of acute‐phase proteins, as well as by increased levels of pro‐inflammatory cytokines.^40^ Microglia‐mediated neuroinflammation is a prominent feature shared by various neurodegenerative diseases and mental illness.^13^, ^41^ Upregulation of lncRNA HOXA‐AS2 promotes neuroinflammation by regulating microglial polarization through interacts with the polycomb repressive complex two complex and epigenetically silencing peroxisome proliferator‐activated receptor gamma coactivator‐1a.^13^ Therefore, HOXA‐AS2 may also promote neuroinflammation in the BD patients. In this study, we have seen a significant elevation in HOXA‐AS2 levels in patients with BD compared to individuals without the condition. This finding is being reported for the first time.

MEG3 is a gene that is only expressed when inherited from the mother and is found in various healthy tissues.^42^ This lncRNA plays a role in differentiating GABAergic neurons.^43^ Remarkably, the increase in MEG3 expression leads to an up‐regulation of pro‐inflammatory cytokines, a decrease in oxidative stress and apoptosis, and an enhancement in the survival of hippocampus neurons via activating the PI3K/AKT/mTOR pathway.^44^ Studies have consistently found elevated levels of inflammatory cytokines in people with BD.^45^ The expression of MEG3 lncRNA is decreased in schizophrenia, Huntington's disease, BD and epilepsy.^10^, ^46^ Fallah et al. assessed the levels of six lncRNAs in the peripheral blood of 60 individuals diagnosed with schizophrenia and 60 individuals without any psychiatric disorders. The levels of MEG3 in all cases were markedly elevated compared to the controls.^6^ In line with this research, the increased expression of MEG3 ncRNA in this study could potentially elevate the likelihood of inflammation and immunological dysregulation in BD. Furthermore, elevated levels of MEG3 have been documented in the nucleus of individuals addicted to heroin.^47^ Heroin affects dopaminergic, glutamatergic and GABAergic circuits, which play a role in the pathogenesis of schizophrenia.^48^ Therefore, MEG3 may also affect the above neurotransmitters and play a role in the pathogenesis of BD.

The stark sex‐based contrast in lncRNA expression markers may indicate that these lncRNAs play sex‐specific roles. The significance of the observed variations in lncRNA profiles between male and female BD patients becomes more prominent when considering the substantial disparities in genetic markers inside the adult brain based on sex. Furthermore, specific genes that exhibit differential expression between sexes are linked to diseases and may possess functional importance. Typically, the presence of sex‐specific gene regulatory systems is shown by sex‐specific expression.^49^ HOXA‐AS2 overexpression was not significantly different between male and female cancer patients.^50^ Maloum et al. was shown that the expression level of *MEG3* were significantly decreased in male BD patients compared to male controls.^10^ This study discovered a substantial difference in the expression of HOXA‐AS2 between male BD patients and controls, as well as a difference in the expression of MEG3 between female BD patients and controls.

This investigation is the first to examine the expression of lncRNAs HOXA‐AS2 and MEG3 in peripheral blood to find possible biomarkers for BD. Our findings indicate that the levels of HOXA‐AS2 and MEG3 lncRNA in peripheral blood have the potential to be effective biomarkers. Compared with the other databases, the starBase provides the most comprehensive miRNA‐lncRNA interactions to date. It was predicted that HOXA‐AS2 was targeted by 19 miRNAs, and MEG3 was targeted by four miRNAs.

There are limitations that also should be considered in this study, including restricted study population (Iran), the sample size used in the study, and many molecular and socio‐cultural variables that can altogether influence the expression level of these genes.

## 5 CONCLUSION

The findings indicate that the HOXA‐AS2 and MEG3 lncRNAs are probably linked to a higher susceptibility to type I BD through several molecular processes. Nevertheless, it is premature to make definitive inferences. Our data demonstrate, for the first time, that the levels of these transcripts can serve as a promising diagnostic signal for persons with BD. Nevertheless, further extensive and prolonged investigations are required to validate the significance of this putative biomarker in BD.

## AUTHOR CONTRIBUTIONS

**Maryam Hosseini:** Data curation (equal); investigation (equal). **Mohammad Javad Mokhtari:** Conceptualization (equal); data curation (equal); formal analysis (equal); investigation (equal); methodology (equal); project administration (equal).

## CONFLICT OF INTEREST STATEMENT

The authors declare no conflicts of interest.

## CONSENT

Participate: Written consent was obtained from all participants.

## Data Availability

The datasets are available from the corresponding author upon reasonable request.
